# Investigation of effects of interlayer interaction and biaxial strain on the phonon dispersion and dielectric response of hexagonal boron arsenide

**DOI:** 10.1038/s41598-023-48654-9

**Published:** 2023-12-04

**Authors:** Somayeh Behzad, Raad Chegel

**Affiliations:** 1 Department of Engineering Physics, Kermanshah University of Technology, Kermanshah, Iran; 2 Department of Physics, Faculty of Science, Malayer University, Malayer, Iran

**Keywords:** Electronic properties and materials, Graphene, Nanoscale materials, Optical materials and structures, Condensed-matter physics, Materials for optics, Nanoscale materials, Electrical and electronic engineering

## Abstract

In this study, the effects of interlayer interaction and biaxial strain on the electronic structure, phonon dispersion and optical properties of monolayer and bilayer BAs are studied, using first-principles calculations within the framework of density functional theory. The interlayer coupling in bilayer BAs causes the splitting of out-of-plane acoustic (ZA) and optical (ZO) mode. For both structures, positive phonon modes across the Brillouin zone have been observed under biaxial tensile strain from 0 to 8%, which indicate their dynamical stability under tensile strain. Also, the phonon band gap between longitudinal acoustic (LA) and longitudinal optical (LO)/transverse optical (TO) modes for monolayer and bilayer BAs decreases under tensile strain. An appreciable degree of optical anisotropy is noticeable in the materials for parallel and perpendicular polarizations, accompanied by significant absorption in the ultraviolet and visible regions. The absorption edge of bilayer BAs is at a lower energy with respect to the monolayer BAs. The results demonstrate that the phonon dispersion and optoelectronic properties of BAs sheet could as well be tuned with both interlayer interaction and biaxial strain that are promising for optoelectronic and thermoelectric applications.

## Introduction

The distinctive physical properties and potential applications of two-dimensional (2D) materials, including graphene, hexagonal BN, MoS_2_, and phosphorene, have garnered significant attention^1–5^. However, these 2D materials have inherent weakness in certain aspects. Graphene shows high electron mobility, which makes it particularly attractive for applications in sensors^6^ and photovoltaic cells^7^. However, the absence of a notable band gap in graphene restricts its potential application in digital electronics^8^. The BN monolayer is a semiconductor that possesses high chemical and thermal stability, but it has an excessively large band gap of about 6 eV and behaves nearly as insulator which cannot function as switch in transistor devices^9–11^.

Monolayer MoS_2_ and phosphorene have suitable band gaps of about 1.8 eV and 1.5 eV, respectively^12,13^. Nevertheless, the carrier mobility of MoS_2_ is only 200–500 cm^2^/(V s), which is lower the carrier mobility of graphene, thereby limiting its application in nanoelectronic devices^14,15^. The phosphorene-based field effect transistor (FET) devices have larger hole mobility of order 10^4^ cm^2^ V^−1^ s^−1^ have been given, but the devices are chemically instable and easy to degrade^16–18^. Therefore, it is significant and necessary to develop novel 2D materials with moderate band bap, high carrier mobility, and high thermal and chemical stability.

Recently, other 2D honeycomb structure of III–V binary compounds, BX (X = BP, BAs, and BSb) monolayers have attracted intensive research interests^19,20^. These systems are found to be dynamically stable as predicted by the calculated phonon dispersion spectrum^9^. Density functional theory calculations have shown that the BP, BAs, and BSb monolayers possess direct band gaps around 1.0 eV^9,21,22^. Xie et al. showed that monolayer BP, BAs, and BSb also show high carrier mobilities exceeding 10^4^ cm^2^ V^−1^ s^−1^, which is comparable to the high carrier mobility of graphene^23^. Therefore, BP, BAs, and BSb monolayers with high carrier mobility, which exceeds than that of phosphorene, and suitable direct band gap are promising channel materials for the production of next-generation 2D FET.

Researchers have synthesized bulk zinc-blende BAs and confirmed its properties through both theoretical predictions and experimental verifications^24–26^. At room temperature, it has been discovered that zinc-blende BAs exhibits a high thermal conductivity of approximately 1300 W m^-1^ K^-1^^27^, semiconducting band structure with a suitable bandgap between 1.5 and 2.0 eV^25,28,29^, and good mechanical performance^30^. Moreover, among all the III–V compounds, BAs is the most covalent III–V compound^31^ and it is stable against chemical decomposition^32^. These intriguing properties motivated researchers to investigate electro-optical, thermal, and transport characteristics of BAs sheets in greater depth^20,33–36^. The studies have shown that BAs monolayer exhibits semiconducting properties with a direct band gap, which can be modulated by applying the strain and it is dynamically stable, as evidenced by the phonon mode behavior^37,38^. The h-BAs monolayer has a smaller bandgap than other III-V group materials such as BN, AlN, and BP monolayer. This suggests that the BAs monolayer has a higher electrical conductivity than these other materials, which could make it a promising material for applications in alkali metals-ion batteries^39^. The BAs monolayer is a promising material for ideal alloy systems due to its greatest covalent character, stability against chemical decomposition and dissolution, and well-aligned valence bands^40–43^. In addition, BAs monolayer possess high carrier mobility, high thermal conductivity comparable to graphene, and exhibits photoactivity under illumination by visible and UV light^23,38,44,45^. Research on the electrical transport properties of hexagonal BAs has revealed behaviors suitable for short-wavelength optoelectronic devices^38^. Theoretical studies have shown that BAs sheet has the potential to be used as gas sensor with high sensitivity and selectivity^35,46^. The combination of these useful properties makes BAs monolayer a promising 2D material for next-generation photonic and electronic applications.

Applying the strain on 2D materials is a highly effective method for modifying their properties^47–50^. By deforming the lattice parameters of a 2D material, strain can alter the electronic, optical and phononic properties of the material in various ways. For instance, strain can induce changes in the bandgap, carrier mobility, and thermal conductivity of a 2D material, leading to enhanced or new functionalities. The impact of strain on the phonon dispersion can affect the thermal conductivity of the material.

Bilayer systems are of great interest as they offer additional control over properties compared to monolayers, through the interlayer interactions which can be tuned via stacking orientation, interlayer spacing, external electric fields, etc.^51–53^. In this paper, we investigate the phonon dispersion, electronic, and optical properties of bilayer BAs under strain, which has not been done before. By comparing the results with the corresponding monolayer cases, we provide a comprehensive description of the physical outcomes.

## Computational model and method

We have used density functional theory (DFT) as implemented in SIESTA^54^ code to study the electronic and optical properties. For exchange–correlation functional, the Perdew-Burke-Ernzerhof (PBE) type of the generalized gradient approximation (GGA) was implemented^55^. In order to determine band gaps with higher accurately, we have used the Heyd–Scuseria–Ernzerhof (HSE06) hybrid functional with the HONPAS package^56,57^. The selection of the double zeta polarized (DZP) basis set was accompanied by setting the orbital confining cut-off at 0.01 Ry. The value of the mesh cutoff for real space projection is 500 Ry. The threshold value for the total energy and forces during structural optimization is set to 10^-6^ eV and 0.01 eV/Å, respectively. The first Brillouin zone is sampled with a 10 × 10 × 1 Monkhorst–Pack grid of k-points. For optical properties, a sufficiently dense k-point grid of 150 × 150 × 1, within the Monkhorst–Pack scheme is used. Optical broadening of 0.15 eV was used for optical spectra.

To structural optimizations and phonon dispersion calculations were performed using the JDFTx package. Incorporation of the van der Waals (vdW) interaction was achieved by implementing the dispersion correction through the use of the DFT-D2 method^58^. The DFT calculations with a plane-wave basis set with a 50-hartree kinetic energy cutoff and, throughout, Coulomb truncation is included. During the process of structural optimizations, the relaxation of atoms was carried out until the force acting on each atom reached a convergence value of 0.1 mHa bohr^−1^. To capture the phonon properties, a 4 × 4 × 1 supercell is used. To prevent interlayer interactions, a vacant space of 20 Å separates the sheets.

To simulate the in-plane tensile strain, the formula ε = (a − a_0_)/a_0_ is used, where a_0_ represents the unstrained lattice constant and a represents the strained lattice constant.

The absorption coefficient α(ω) and reflectance R(ω) can be calculated by the following formula^59^:

1 $$\alpha \left( \omega \right) = \frac{\sqrt 2 \omega }{c}\left\{ {\left[ {\varepsilon_{1}^{2} \left( \omega \right) + \varepsilon_{2}^{2} \left( \omega \right)} \right]^{1/2} - \varepsilon_{1} \left( \omega \right)} \right\}^{2}$$

2 $$R\left( \omega \right) = \left| {\frac{{\sqrt {\varepsilon_{1} \left( \omega \right) + i\varepsilon_{2} \left( \omega \right)} - 1}}{{\sqrt {\varepsilon_{1} \left( \omega \right) + i\varepsilon_{2} \left( \omega \right)} + 1}}} \right|^{2} .$$

## Results and discussion

The optimized geometric structure of BAs monolayer is similar to graphene where alternating B and As atoms replace carbon atoms in the hexagonal lattice with a space group of P-6m2. The relaxed lattice parameters of BAs are a = b = 3.39 Å with B–As bond length of 1.95 Å and bond angle of 120° which coincide with previous results^19,23,41,60,61^.

**Figure 1:**
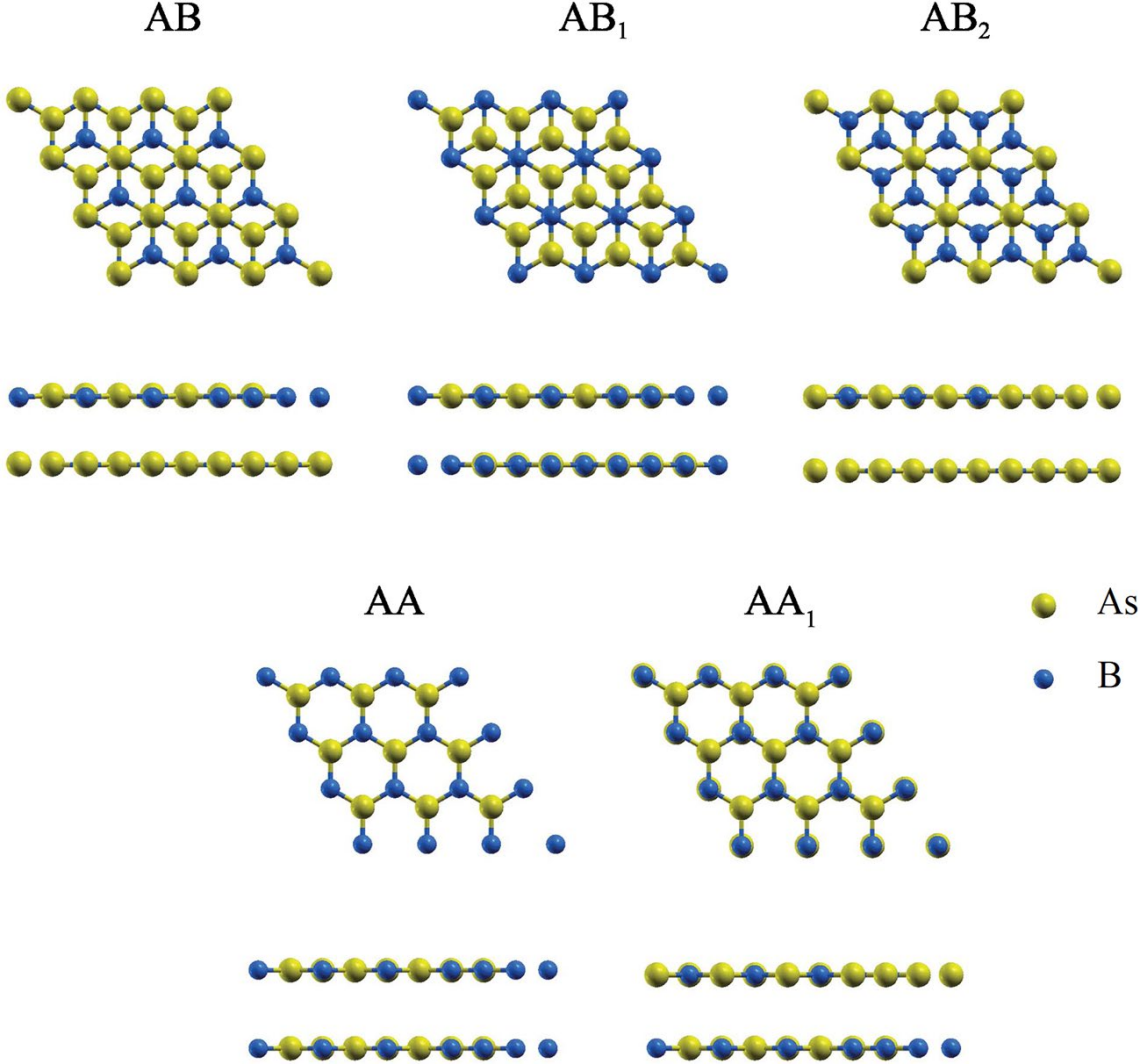
Top and side views of five different stacking patterns of bilayer BAs.

In order to find the most stable stacking mode of the BAs bilayer, five stacking patterns, namely AA, AA_1_, AB, AB_1_ and AB_2_ were considered (see Fig. 1). In the AA (AA_1_) stacking pattern, the B atoms of top layer are located above the B (As) atoms of bottom layer. For AB stacking pattern, the B atoms on top layer are located above the As atoms on bottom layer, while As atoms on top layer centered above the hexagon of the bottom layer. In the AB_1_ (AB_2_) stacking pattern, the B (As) atoms on top layer are located above the B (As) atoms on bottom layer, while As (B) atoms on top layer centered above the hexagon of the bottom layer. After geometry optimizations, the interlayer distances of bilayer BAs for the AA, AA_1_, AB, AB_1_ and AB_2_ stacking patterns are 4.12, 3.78, 3.37, 3.57 and 3.85 Å, respectively. The binding energy (E_b_) was obtained as the difference between the total energy of the BAs bilayers (E_tot_) and the isolated BAs monolayers (E_m_), as follows^40,61,62^:

3 $${\text{E}}_{{\text{b}}} = {\text{E}}_{{{\text{tot}}}} - {\text{2E}}_{{\text{m}}}$$

The calculated binding energy for the AA, AA_1_, AB, AB_1_ and AB_2_ stacking patterns are − 40.55, − 63.86, − 95.51, − 84.48 and − 56.19 meV, respectively. The shortest interlayer distance and the smallest binding energy of the AB stacking pattern confirm that the AB stacking pattern is the most energetically favorable. After the structural optimizations, the planer bilayer structure is converted into buckled one, due to the interlayer interactions. For bilayer BAs, the optimized value of buckling height and B-As bond length is 0.96 Å and 1.96 Å, respectively.

**Figure 2:**
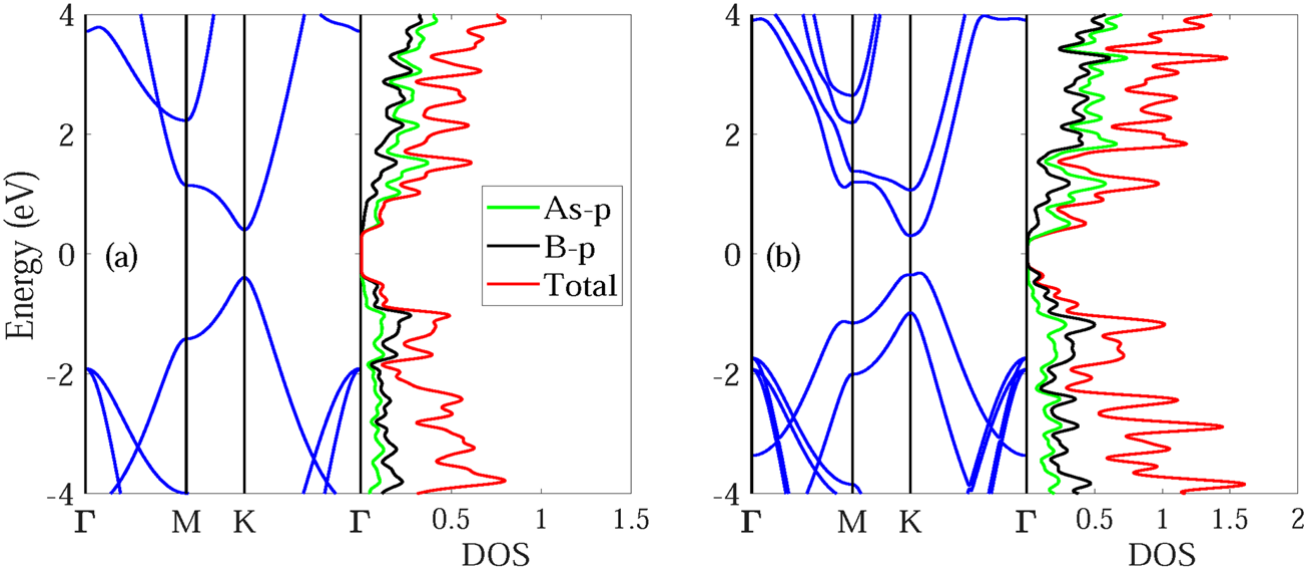
Electronic band structure and correspnding partial density of states (PDOS) for (**a**) monolayer and (**b**) bilayer BAs.

The calculated electronic band structures along with the corresponding partial density of states (PDOS) of monolayer and bilayer BAs is presented in Fig. 2. The BAs monolayer exhibits direct gap semiconducting behavior with a band gap of 0.79 eV using PBE method and 1.19 eV using HSE06 method. Both the conduction band minimum (CBM) and valence band maximum (VBM) of BAs monolayer lie at the K point. The appreciable band gap of BAs could make it suitable for use in nanoelectronics applications and the direct band gap characteristics of this material enables it to have several applications in the optical devices field because of the lower energy required to form excitons.

The bilayer BAs is an indirect gap semiconductor. The CBM is situated at the K point while the VBM is slightly far from the K point. The calculated band gap is 0.63 eV using PBE method and 0.96 eV using HSE06 method. The calculated PDOSs show the contributions of individual orbitals of monolayer and bilayer BAs. The VBM is mainly contributed by p orbitals of As, and CBM mainly comes from the p orbitals of B.

**Figure 3:**
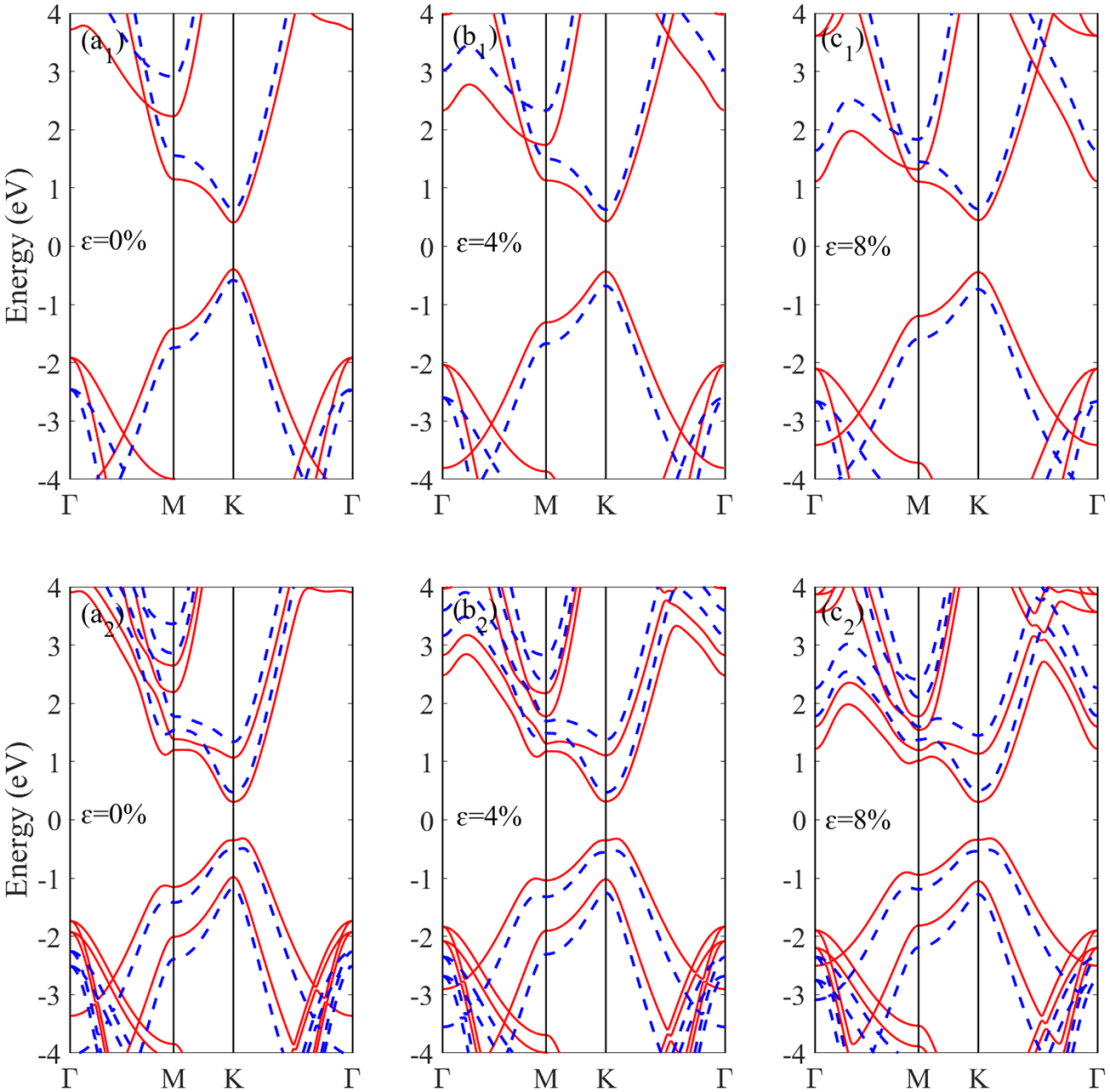
The electronic band structure based on HSE06 (blue dashed) and PBE (red) methods for monolayer BAs under biaxiall strain of (**a**_**1**_) 0%, (**b**_**1**_) 4% and (**c**_**1**_) 8% and bilayer BAs under biaxiall strain of (**a**_**2**_) 0%, (**b**_**2**_) 4% and (**c**_**2**_) 8%.

The calculated band structures of monolayer and bilayer BAs subjected to different biaxial strains is demonstrated in Fig. 3. Up to 8% of strain, the monolayer (bilayer) BAs demonstrates direct (indirect) band gap semiconducting behavior. For both structures, by increasing the tensile strain, conduction band edge at Γ point shifts downward relative to the Fermi level. Applying a strain in the range of 0–8% results in a slight increase of the band gap value of monolayer BAs, from 0.79 (1.19) to 0.89 (1.36) eV using PBE (HSE06) method, while the band gap value of bilayer BAs remains almost unchanged.

**Figure 4:**
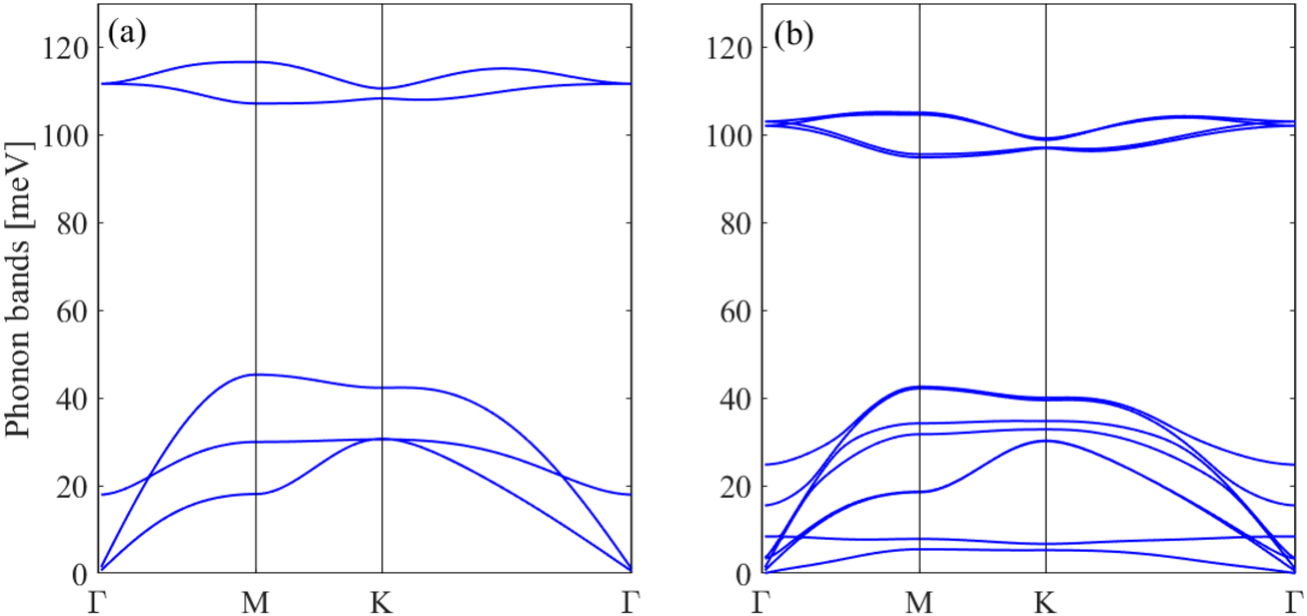
Phonon dispersion spectrum of (**a**) monolayer and (**b**) bilayer BAs.

Figure 4a shows the phonon dispersion of monolayer BAs. No imaginary modes are observed in the first Brillouin zone for monolayer BAs, indicating its dynamical stability. There are three acoustic and three optical phonon modes corresponding to the two atoms in one unit cell. The LA and TA modes exhibit linear dispersions in the vicinity of the Γ point, while the ZA mode has a quadratic dispersion relation because of the 2D character of monolayer BAs. The phonon energy of the longitudinal optical (LO) and transverse optical (TO) degenerate modes at Γ point for BAs is 111 meV. The LO and TO branches are also degenerated near the K point, indicating strong covalent bond between the B and As atoms. Also, a direct phonon gap of 62 meV between the acoustic braches and LO and TO branches is observed.

Figure 4b shows the phonon dispersion of the bilayer BAs. There are 12 phonon modes, including 9 optical modes and 3 acoustical modes in the bilayer structure corresponding to the four atoms in one unit cell. If two layers are placed together without interaction, we expect that their phonon dispersions completely overlap. The phonon dispersion of bilayer BAs is similar to the one of monolayer BAs and most of the branches in the bilayer can be viewed as the ones split from the monolayer. Very small changes are observed in the in-plane doubly degenerated bands (the in-plane LA and LO^1^, TA and TO^1^, LO^2^ and LO^3^, TO^2^ and TO^3^) implying that the main effect of the interlayer interactions is due to the ZA modes. The weak interlayer coupling causes the splitting of out-of-plane ZA and ZO modes to ZA and ZO^1^ and ZO^2^ and ZO^3^. The splitting of ZA and ZO^1^ and ZO^2^ and ZO^3^ has an appreciable magnitude near the Γ point.

**Figure 5:**
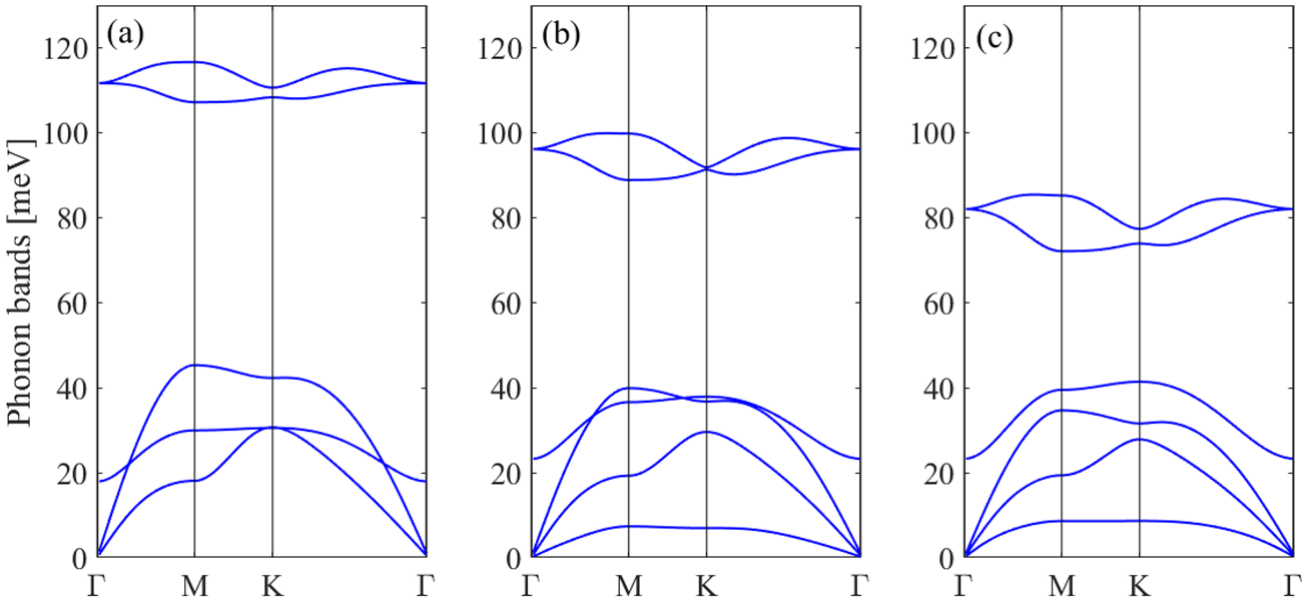
Phonon dispersion spectrum of monolayer BAs under biaxial strain of (**a**) 0%, (**b**) 4% and (**c**) 8%.

**Figure 6:**
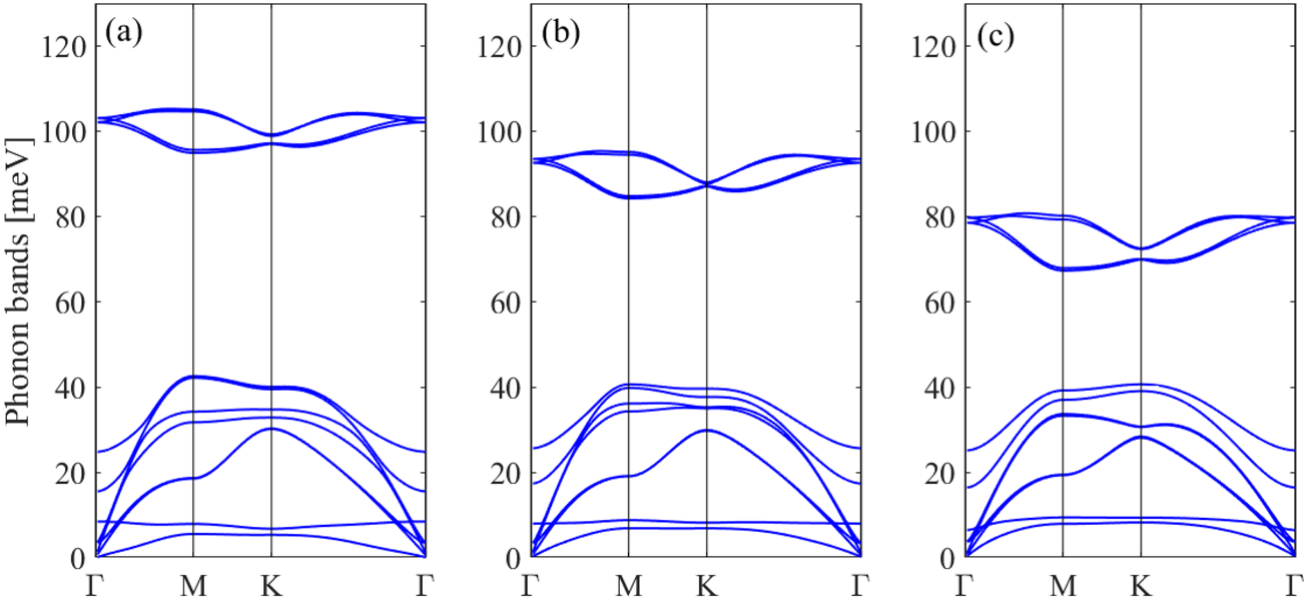
Phonon dispersion spectrum of bilayer BAs under biaxial strain of (**a**) 0%, (**b**) 4% and (**c**) 8%.

Next, we study the effect of in-plane biaxial strain on the phonon dispersion of monolayer and bilayer BAs. The structural optimizations show that the value of buckling is decreased under tensile strain. Figures 5 and 6 display the phonon spectrum of the monolayer BAs under biaxial tensile strain. There are no negative phonon frequencies under tensile strain which indicates that the monolayer and bilayer BAs are dynamically stable under tensile strain.

For monolayer BAs, by increasing the tensile strain the LO/TO, LA and TA phonon modes become soften which can be attributed to the weakness of atomic bond strength by increasing the lattice constant. The phonon band gap between LO/TO and LA modes decreases by increasing the tensile strain. A hardening of the ZO and ZA modes is also found for monolayer BAs under biaxial tensile strain and the hybridization between LA/TA and ZO mode is removed. For all applied tensile strains, the acoustic TA/LA/ZA phonons remain degenerate at Γ-point.

For bilayer BAs, by applying the tensile strain the )LO^2^, LO^3^/TO^2^, TO^3^) phonon modes shift to lower mode frequencies leading to phonon softening and the phonon band gap between (LO^2^, LO^3^/TO^2^, TO^3^) and LA and LO^1^ decreases. The ZA mode becomes harden, similar to the monolayer structure.

In order to examine how interlayer interaction and strain affect the BAs sheet, we calculated the most important optical parameter, namely, the complex dielectric function which is represented by: ε(ω) = ε_1_(ω) + iε_2_(ω) where ε_1_(ω) and ε_2_(ω) are the real and imaginary terms, respectively. By counting the matrix elements of interband optical transitions between occupied and unoccupied states using the random phase approximation (RPA), it is possible to identify the imaginary part. The Kramers–Kronig relations can then be applied to compute the real part. With both the real and imaginary components, it becomes feasible to calculate other optical parameters of a material, including its absorption and reflectivity spectra.

**Figure 7:**
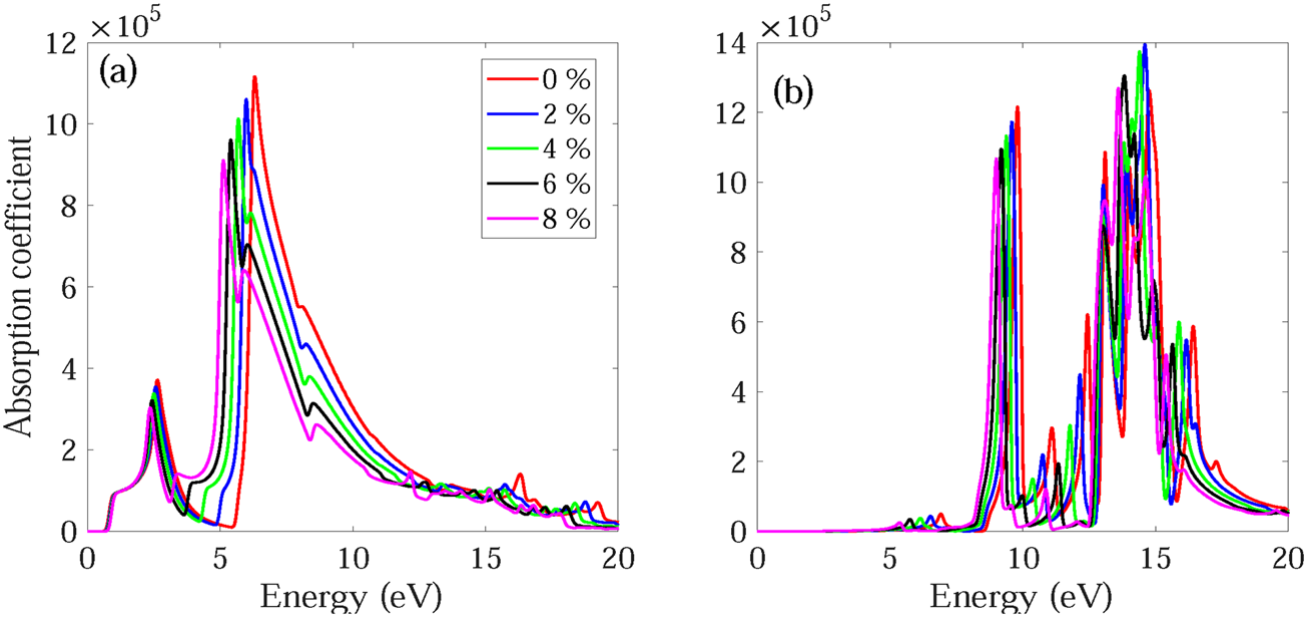
Calculated absorption coefficient α(ω) for light polarized along the (**a**) x and (**b**) z directions for monolayer BAs under biaxial tensile strain.

**Figure 8:**
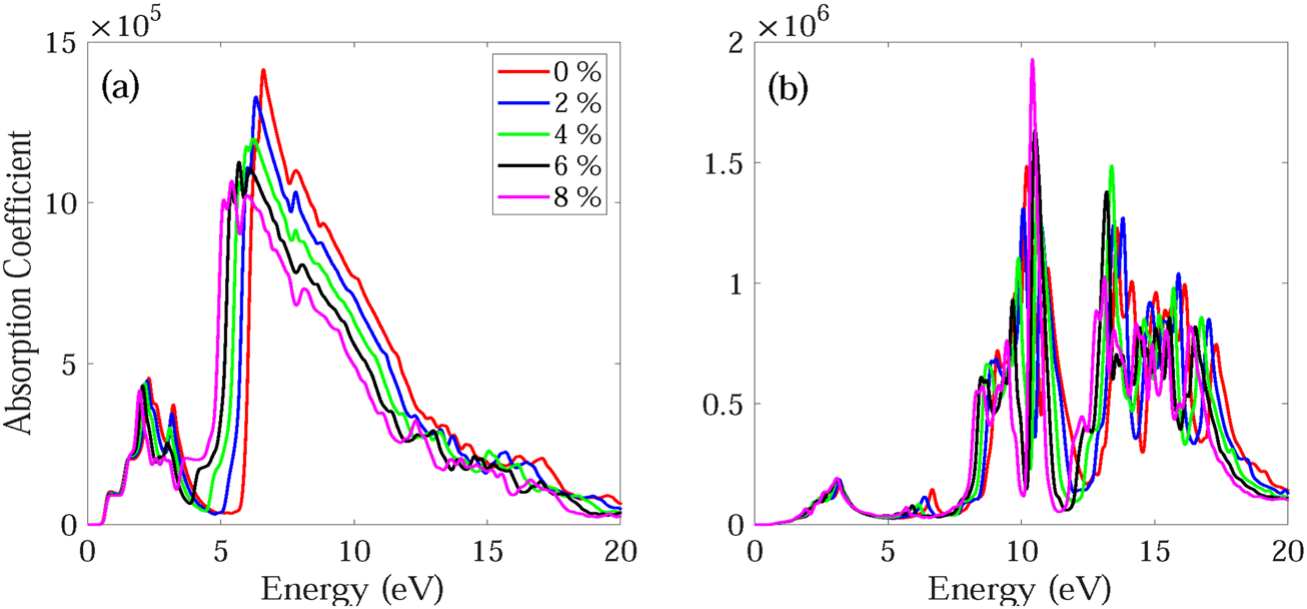
Calculated absorption coefficient α(ω) for light polarized along the (**a**) x and (**b**) z directions for bilayer BAs under biaxial tensile strain.

**Figure 9:**
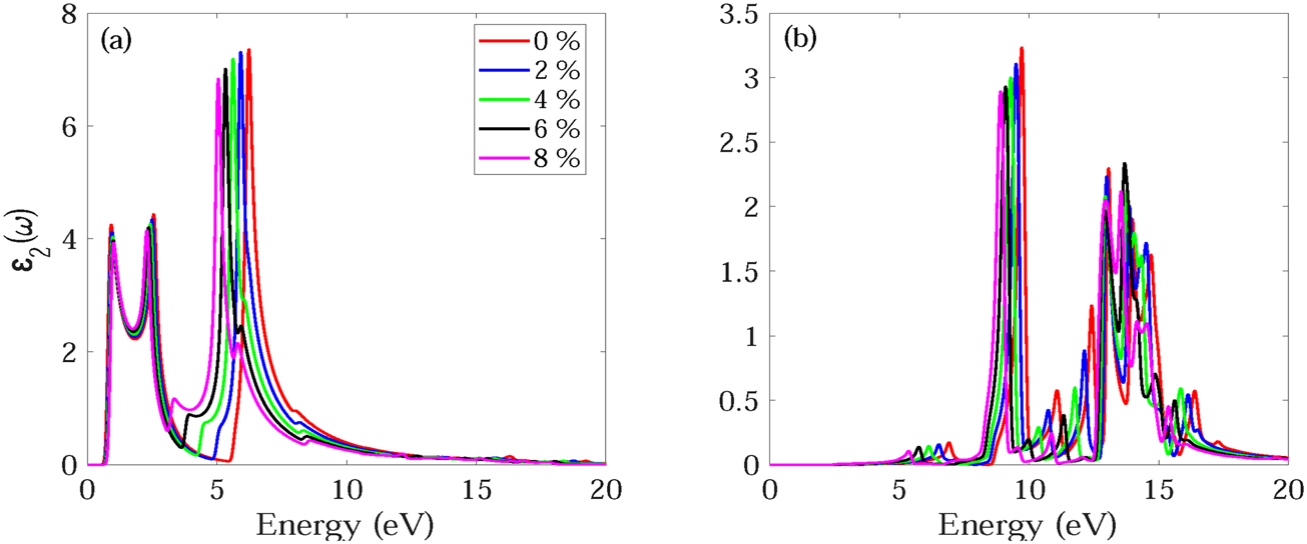
Imaginary part ε_2_(ω) of dielectric function for light polarized along the (**a**) x and (**b**) z directions for monolayer BAs under biaxial tensile strain.

**Figure 10:**
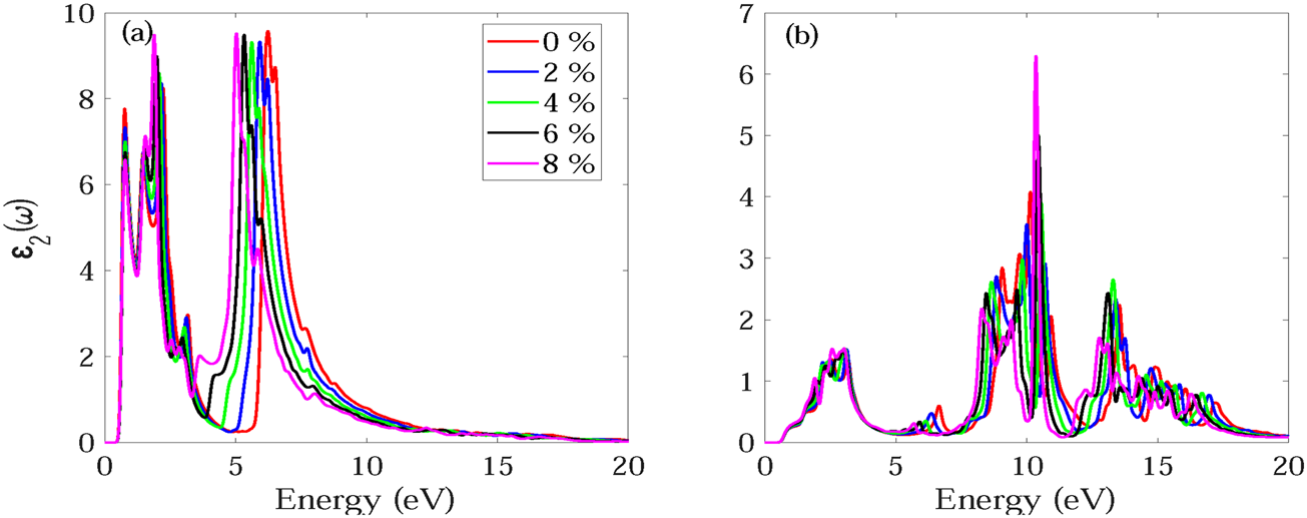
Imaginary part ε_2_(ω) of dielectric function for light polarized along the (**a**) x and (**b**) z directions for monolayer BAs under biaxial tensile strain.

**Figure 11:**
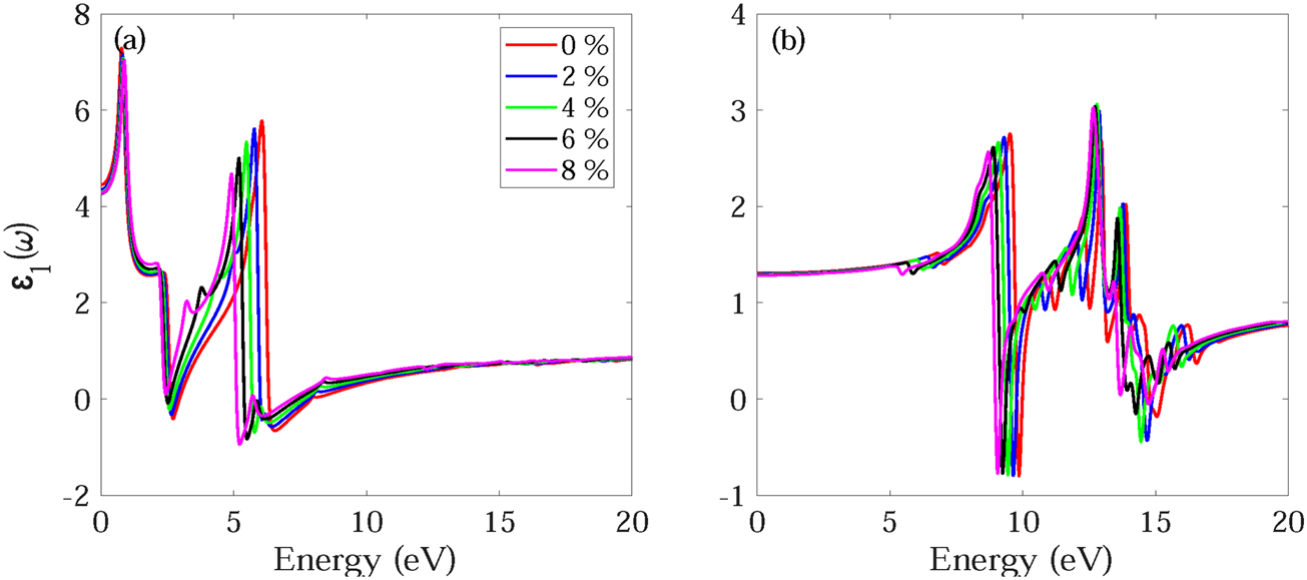
Real part ε_1_(ω) of dielectric function for light polarized along the (**a**) x and (**b**) z directions for monolayer BAs.

**Figure 12:**
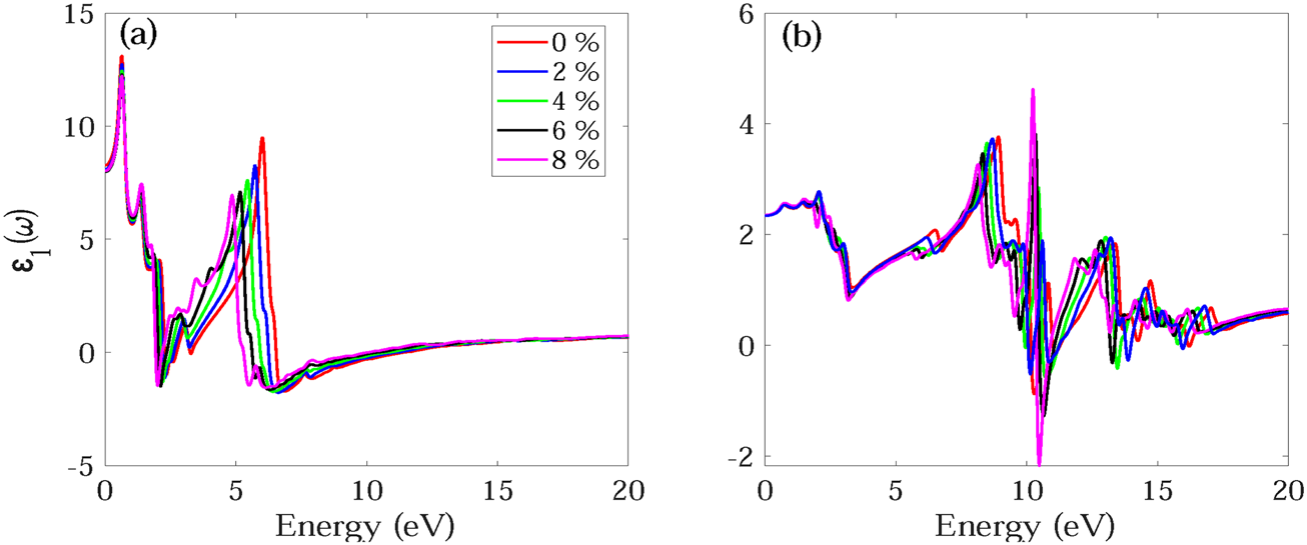
Real part ε_1_(ω) of dielectric function for light polarized along the (**a**) x and (**b**) z directions for bilayer BAs.

**Figure 13:**
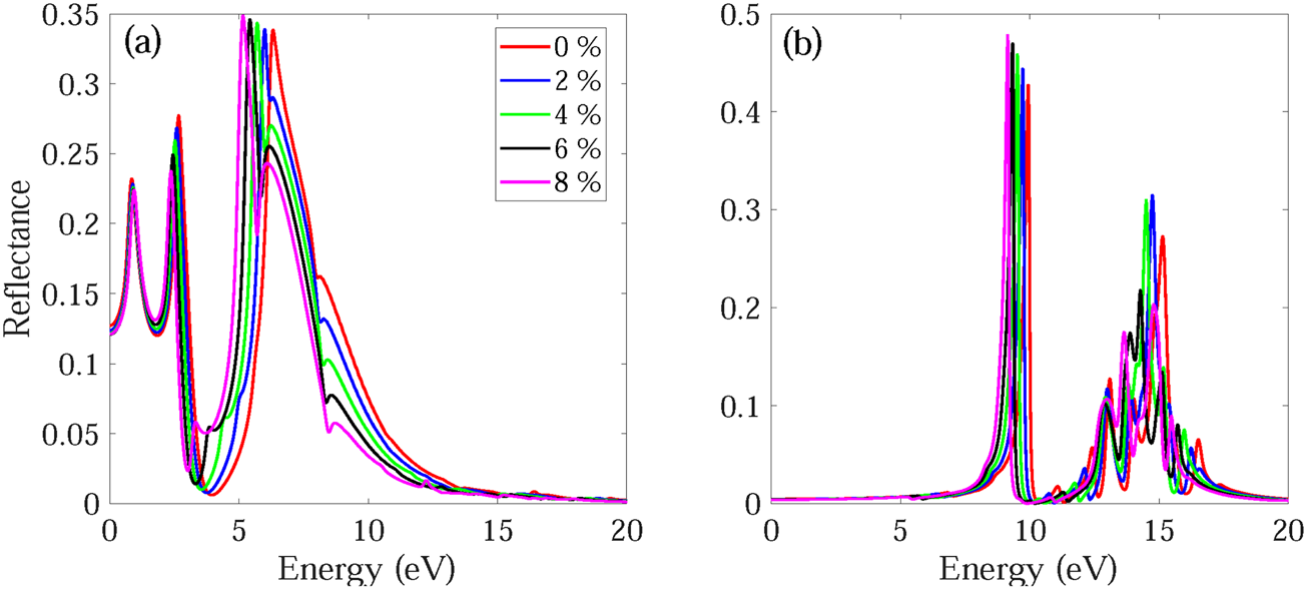
Reflectance spectra R(ω) for light polarized along the (**a**) x and (**b**) z directions for monolayer BAs under biaxial tensile strain.

**Figure 14:**
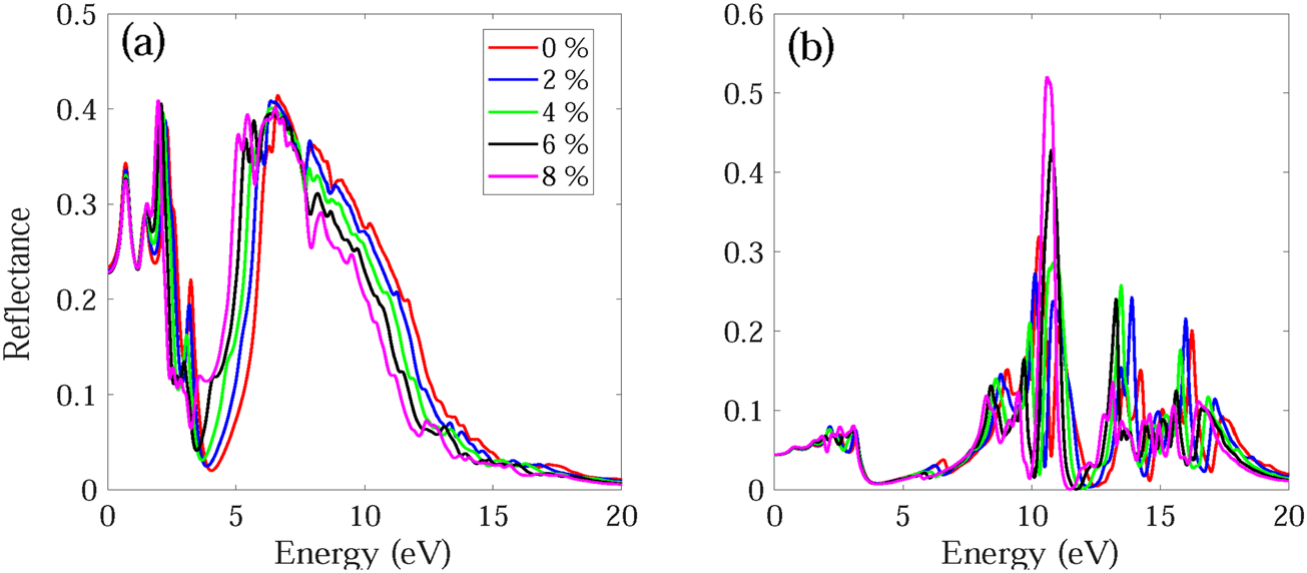
Reflectance spectra R(ω) for light polarized along the (**a**) x and (**b**) z directions for bilayer BAs under biaxial tensile strain.

The frequency dependent absorption coefficient α(ω), real ε_1_(ω) and imaginary part ε_2_(ω) of dielectric function, and reflectance R(ω) for monolayer and bilayer BAs without and with strain are investigated and demonstrated in Figs. 7, 8, 9, 10, 11, 12, 13, 14. Anisotropy of optical spectra for parallel and perpendicular polarizations is obvious from Figs. 7, 8, 9, 10, 11, 12, 13, 14. The anisotropy of the optical spectra can be clearly observed for parallel and perpendicular polarizations as depicted in Figs. 7, 8, 9, 10, 11, 12, 13, 14.

Figures 7 and 8 show the absorption spectrum of monolayer and bilayer BAs under biaxial tensile strain, respectively. For light polarized along the x direction, the unstrained monolayer BAs shows a pronounced absorption in the UV and visible regions. In particular, a strong peak is observed at 6.32 eV, while a weaker peak is visible at 2.66 eV within the visible region. The absorption spectrum of unstrained bilayer BAs contains some shoulders and additional weak peaks with respect to the monolayer BAs. The bilayer BAs has two main peaks at 2.30 and 6.59 eV, respectively (see Fig. 8a). The absorption onset energies for monolayer and bilayer BAs are observed to be approximately 0.69 eV and 0.55 eV, respectively. These values are consistent with the presence of a direct band gap at the K point, with corresponding energies of 0.79 eV and 0.63 eV for monolayer and bilayer BAs, respectively. The shift of the absorption edge and first main absorption peak to lower energies in bilayer BAs is due to the interlayer interactions. These findings suggest that the absorption of light by BAs occurs through the excitation of electrons from the valence band to the conduction band, and that this process is more effective in bilayer BAs compared to monolayer BAs.

When light is polarized along the z direction, the unstrained monolayer BAs sheet exhibits an almost negligible absorption in the visible region. However, it demonstrates a robust absorption feature in the UV region with significant absorption peaks at 6.92, 9.80, 11.11, 12.45, 13.10, 14.76, and 16.42 eV. The absorption spectrum of the unstrained bilayer BAs has a small peak at visible region and several absorption peaks at UV region. The unstrained bilayer BP has main absorption peaks at 3.22, 6.67, 10.19, 13.62, 16.15 and 17.32 eV. It can be observed that the number of peaks in the bilayer structure is more than the monolayer structure and the peaks become broader, due to the interaction between the layers along the z direction.

The absorption spectra of monolayer and bilayer BAs show an effective absorption of light in both the visible and UV regions. This property makes them promising candidates for the development of optoelectronic devices that operate at short wavelengths, where high absorption efficiency is critical. Additionally, their ability to absorb UV light could make them useful in applications where protection against UV radiation is important, such as in sunscreen or protective coatings. Overall, the demonstrated ability of BAs to absorb light in multiple regions of the electromagnetic spectrum highlights their potential for a range of applications in the field of materials science and optoelectronics.

The calculated imaginary part ε_2_(ω) of dielectric function for monolayer and bilayer BAs under biaxial tensile strain is demonstrated in Figs. 9 and 10. For light polarized along the x direction, the ε2(ω) for monolayer BAs shows three significant peaks at 0.92, 2.56 and 6.24 eV in the near-IR, visible, and UV regions of the electromagnetic spectrum, respectively. For bilayer BAs, there are two strong optical absorption regions with multiple peaks in the range 0.6–3.4 and 5.8–9.2 eV. The main absorption peaks occur at 0.76, 1.49, 2.23 and 6.24 eV. It can be observed that monolayer BAs exhibits no ε_2_(ω) peak up to 6 eV when subjected to z-direction polarization, which suggests that the material has no optical absorption within this spectral range. For bilayer BAs, a multiple peak is observed in the visible region from 1.9 to 3.4 eV.

The real part of the dielectric function, ε_1_(ω), provides insight into both the electronic polarizability and dispersion effects of the material. Figures 11a and d and 12a and b show ε_1_(ω) for light polarized along the x and z directions for monolayer and bilayer BAs, respectively. The key parameter of interest in this part is the static dielectric constant, which is determined by the magnitude of ε_1_(ω) at ω = 0. The static dielectric constant of the unstrained monolayer BAs has been calculated to be 4.45 and 1.31 along the x and z directions, respectively. The calculated ε_1_(0) for bilayer BAs, is found to be 8.26 and 2.23, along the x and z directions.

The calculated reflectance spectra R(ω) for monolayer and bilayer BAs under biaxial tensile strain is demonstrated in Figs. 13 and 14. For light polarized along the x and z directions, the reflectance spectra static values are observed at some low values of 0.13 and 0.01 (0.23 and 0.04) for monolayer BAs (bilayer BAs), respectively. For light polarized along the x direction, the main reflectance peaks occur at 0.84 , 2.66 and 6.32 eV (0.71, 1.46, 2.32, 3.24 and 6.64 eV) for monolayer BAs (bilayer BAs) with a reflectance of about 23.1%, 27.7% and 33.9% (34.3%, 29%, 38.1% and 41.4%), respectively. These results are consistent with the calculated ε_2_(ω) values.

For light polarized along the x direction, the main absorption peaks of monolayer and bilayer BAs become red-shifted and their intensities decreases as the applied strain changes from ε = 0% to ε =  + 8%. Also, for monolayer and bilayer BAs the first peak of ε2(ω) becomes blue-shifted, while the second and third peaks become red-shifted as the strain changes from ε = 0 to ε =  + 10%. Similar behavior is observed in the reflectance spectrum. The absorption edge for monolayer and bilayer structures remains almost unchanged under application of tensile strain. Therefore, application of compressive strain decreases the absorption of monolayer BAs by decreasing the intensity and width of absorption. The calculated ε_1_(ω) and ε_2_(ω) for monolayer and bilayer BAs using HSE06 method are shown in Figs. S1 and S2.

## Conclusion

Using the first-principles calculations based on DFT, the impact of interlayer interaction and biaxial strain on the band structure, phonon dispersion and optical characteristics of BAs sheet was systematically studied. Based on the phonon dispersion curve calculations, both the monolayer and bilayer BAs are dynamically stable under zero strain. The phonon dispersion of bilayer BAs is very similar to the one of monolayer BAs. Almost no change is observed in the in-plane doubly degenerated bands while, the interlayer coupling causes the splitting of out-of-plane modes. When the tensile strains is applied, both the monolayer and bilayer structures are dynamically stable. For light polarized along the x direction, the main absorption peaks of monolayer and bilayer BAs become red-shifted and their intensities decreases by increasing the tensile strain. This study implies the potential applications of BAs sheet for optoelectronic and thermoelectric devices in nanoscale.

### Supplementary Information

Supplementary Figures.

## Data Availability

The datasets used and analyzed during the current study available from the corresponding author on reasonable request.
