# Supplementary material for: Investigation of effects of interlayer interaction and biaxial strain on the phonon dispersion and dielectric response of hexagonal boron arsenide
Source: Sci Rep. 2023 Dec 4;13:21339. doi: 10.1038/s41598-023-48654-9 (PMC10695960; doi:10.1038/s41598-023-48654-9)
Supplement: Supplementary file 1 — Supplementary Figures. [file 41598_2023_48654_MOESM1_ESM.docx]

Investigation of effects of interlayer interaction and biaxial strain on the phonon dispersion and dielectric response of hexagonal boron arsenide

Somayeh Behzad^*1^, Raad Chegel ^2^

^1^Department of Engineering Physics, Kermanshah University of Technology, Kermanshah, Iran

^2^Physics Department, Faculty of Science, Malayer University, Malayer, Iran


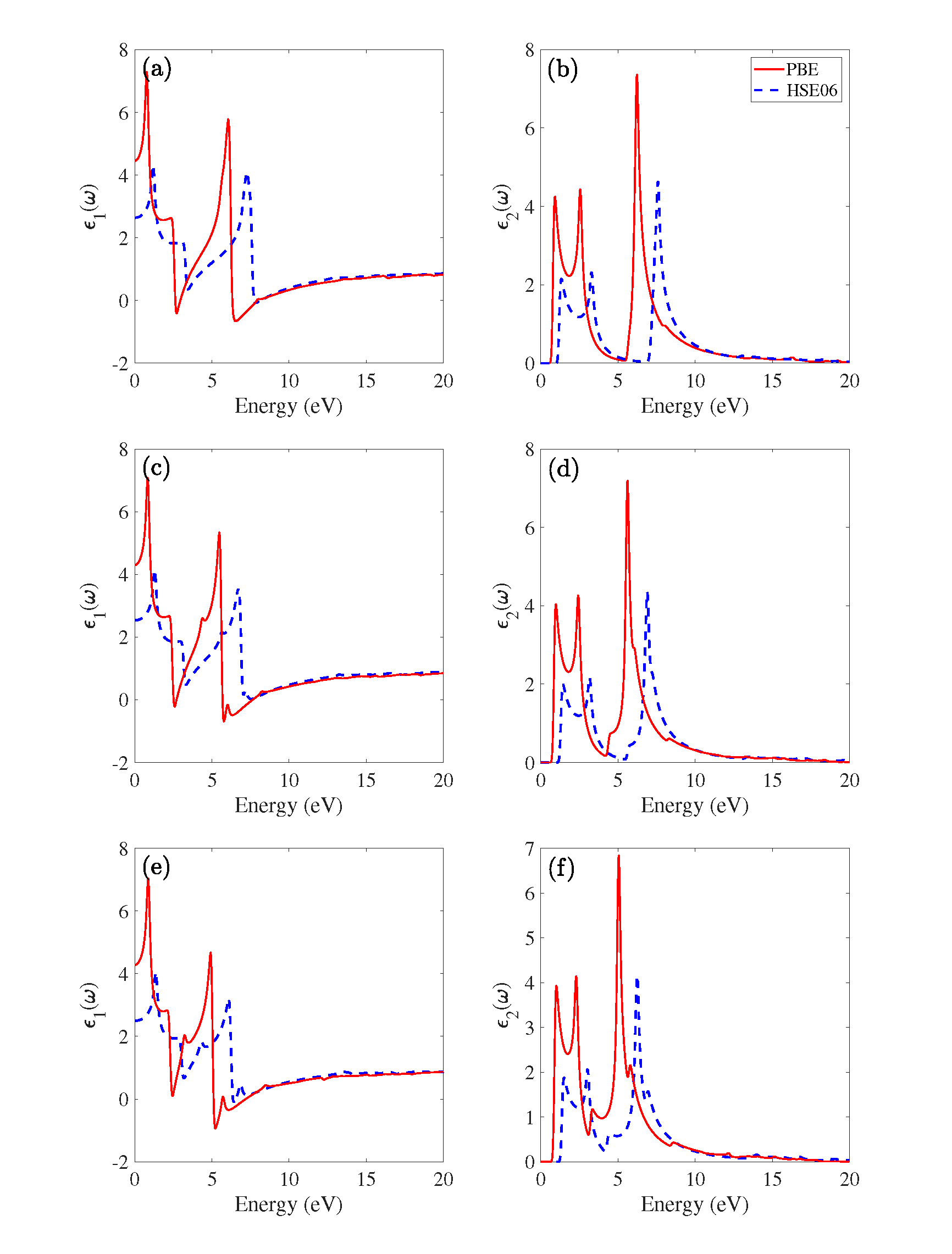


Figure S_1_: The calculated ε_1_(ω) and ε_2_(ω) for monolayer BAs using PBE and HSE06 methods.


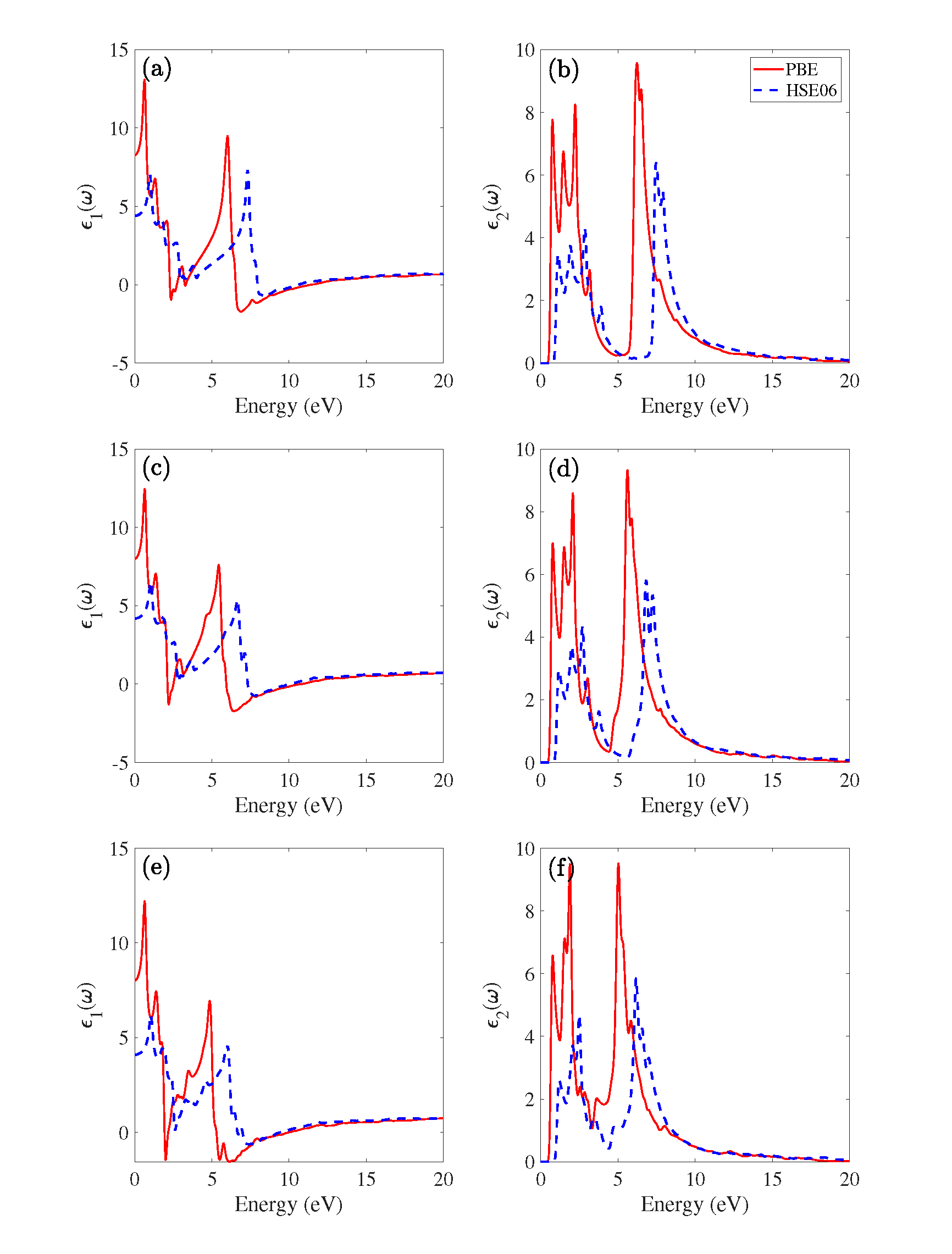


Figure S_2_: The calculated ε1(ω) and ε2(ω) for bilayer BAs using PBE and HSE06 methods.
